# The impact of learning motivation on academic performance among low-income college students: the mediating roles of learning strategies and mental health

**DOI:** 10.3389/fpsyg.2025.1639375

**Published:** 2025-09-11

**Authors:** Yongrong Huang, Yu Li, Guanglei Chen

**Affiliations:** ^1^School of Teacher Education, Heze University, Heze, China; ^2^School of Foreign Languages, Heze University, Heze, China

**Keywords:** intrinsic motivation, extrinsic motivation, amotivation, learning strategies, perceived stress, low-income students, grade-point average

## Abstract

Financial hardship can hinder university achievement, yet the mechanisms linking motivation to performance among low-income undergraduates remain underexplored. This two-wave study tracked 316 students from three public universities in eastern China over a six-month period. Measures included intrinsic, extrinsic, and amotivation, learning strategies, perceived stress, and grade point average (GPA). Structural equation modeling revealed that the proposed model explained over 64% of the variance in GPA. Intrinsic motivation was positively associated with GPA both directly and indirectly through greater use of effective learning strategies and lower perceived stress. Extrinsic motivation contributed indirectly via strategy use, whereas amotivation showed the strongest negative relationship with GPA, operating through both reduced strategy use and elevated stress. These findings suggest that the quality of motivation shapes academic performance through self-regulated learning and stress appraisal. Interventions that foster intrinsic motivation, strengthen metacognitive and analytical strategies, and reduce stress may help narrow achievement gaps for economically disadvantaged students.

## 1 Introduction

Education plays a pivotal role in determining an individual’s life trajectory (Jongbloed et al., 2024), yet students from low-income families often confront structural (Carter et al., 2023) and psychological barriers (Brummelman and Sedikides, 2023) that impede academic success. These challenges frequently stem from limited access to educational resources (Masonbrink and Hurley, 2020), reduced parental involvement, and heightened exposure to environmental stressors, all of which can erode both academic engagement and achievement. Among the many determinants of scholastic performance, learning motivation stands out as a central driver: it not only sustains effort but also shapes the adoption of effective learning strategies and resilience in the face of obstacles (Liu et al., 2023). Empirical evidence consistently shows that students from higher socioeconomic backgrounds outperform their lower-SES peers, a disparity partially explained by differences in academic mindsets. For instance, analysis of nationally representative U. S. high school data revealed that students from higher-SES families were less likely to endorse fixed beliefs about intelligence, with the gap between SES groups reaching 0.22 standard deviations (Destin et al., 2019), suggesting that mindset-related differences may influence both motivational processes and strategic learning behaviors. Similarly, research indicates that family socioeconomic status positively predicts Internet self-efficacy (*β* = 0.20), which in turn facilitates active information searching (*β* = 0.33), formal inquiry (*β* = 0.27), and informal inquiry (*β* = 0.24), thereby indirectly supporting academic help-seeking behaviors (Fan, 2025). Such findings highlight that socioeconomic advantages can bolster motivational and cognitive resources—such as self-efficacy and adaptive learning strategies—that, alongside mental health, may serve as critical mediating mechanisms in the academic outcomes of low-income college students.

Li X. et al. (2023) investigated the effects of family income on academic performance and non-cognitive skills in an economically disadvantaged region of China, finding that low-income students not only achieved lower academic outcomes but also exhibited weaker growth mindset, despite stronger perseverance. Path analysis indicated that growth mindset mediated the income–achievement link negatively, while perseverance mediated it positively, though not enough to counterbalance the disadvantages (Li L. et al., 2023). Zhao et al. (2022) further showed that both family and school SES were positively associated with academic achievement, with parental occupational status and education emerging as the strongest predictors. These effects were moderated by the school’s disciplinary climate, underscoring the role of contextual factors in shaping SES–achievement relationships(Zhao et al., 2022). In the study by Swanson et al. (2021), psychosocial variables were found to be moderately predictive of academic outcomes, with academic self-efficacy and sense of belonging emerging as the strongest predictors of both cumulative GPA and student persistence (Swanson et al., 2021). Boman and Wiberg (2024), drawing on Sweden’s PISA 2018 data, found that both SES and non-cognitive variables—including students’ reading self-concept and growth mindset—significantly predicted achievement in mathematics and reading (Boman and Wiberg, 2024).

Low-income students are often burdened by financial constraints, which can lead to stress, decreased access to resources, and diminished opportunities for academic enrichment (Bolen and Martin, 2005; Morris-Perez et al., 2023). These factors may affect their motivation levels and, ultimately, their academic outcomes (Carroll et al., 2023; Hodis et al., 2011; Scales et al., 2020). Therefore, understanding the relationship between learning motivation and academic performance in this demographic is crucial for informing educational interventions and policies aimed at improving their academic success. The current body of literature on learning motivation and academic performance primarily explores general student populations (Furrer and Skinner, 2003; Kusurkar et al., 2011; Steinmayr et al., 2019), with a notable focus on the intrinsic extrinsic motivation dichotomy (Ginsburg and Bronstein, 1993; Hanus and Fox, 2015). In contrast, few studies have specifically examined how these motivational factors influence the academic performance of low-income students. Moreover, the mechanisms underlying this relationship, such as the potential mediating effects of study strategies or psychological well-being, remain poorly udestood (Geppert et al., 2023; Paauwe, 2008; Plakhotnik et al., 2021). Some researchers argue that in-trinsic motivation is more strongly correlated with academic success than extrinsic motivation (Gao et al., 2023; Kotera et al., 2021) while others suggest that extrinsic rewards, such as scholarships or job opportunities, can significantly enhance performance, particularly for students facing financial difficulties. However, merit-based scholarships in many institutions require not only high academic standing but also costly qualifications such as standardized English proficiency tests (e.g., IELTS or TOEFL) (Jones-Darnell, 2023). For low-income students, these requirements can impose additional financial and psychological burdens, thereby restricting access to crucial academic incentives and potentially dampening their motivation and performance (Ryan, 2023).

This study seeks to fill this gap by using structural equation modeling (SEM) to examine the relationship between learning motivation and academic performance among low-income university students. By considering both intrinsic and extrinsic motivation, as well as potential mediators such as learning strategies and psychological well-being, this research aims to provide a more comprehensive understanding of the factors influencing academic outcomes in this vulnerable group. The findings of this study could have significant implications for educational practices and interventions designed to support low-income students, ultimately helping to mitigate the academic disparities associated with financial hardship.

The primary aim of this study is to explore the direct and indirect relationships between learning motivation and academic performance in low-income university students. It also aims to identify potential mediating factors, such as learning strategies and psychological health, that may explain the observed effects. The principal conclusion drawn from this research will be that enhancing learning motivation, particularly through targeted interventions that address both intrinsic and extrinsic factors, can significantly improve academic performance among low-income students.

### 1.1 Research background and significance

College students from low-income families represent a critical subgroup within higher education contexts, as their academic success significantly affects their long-term socioeconomic mobility and psychological well-being (Dover et al., 2020) Students from eco-nomically disadvantaged backgrounds often face unique barriers, including limited access to educational resources (Jackson and Persico, 2023), financial stress (Chin et al., 2024; Iantosca et al., 2024), and insufficient social support (Stearns et al., 2024), which collectively may impede their academic performance (Lederer et al., 2020; Jenkins et al., 2020) and psychological adaptation (Jenkins et al., 2020). Given these challenges, it is essential to understand the factors and underlying mechanisms that influence the academic outcomes of low-income college students. Among these factors, learning motivation has been consistently highlighted as a pivotal de-terminant of academic achievement (Liu et al., 2013; Shofiah et al., 2023; Wibrowski et al., 2016).

### 1.2 Learning motivation and academic performance

Learning motivation, generally conceptualized as an internal drive directing stu-dents’ engagement, effort, and persistence in educational tasks, includes both intrinsic motivation (engagement driven by inherent interest) and extrinsic motivation (engagement driven by external rewards or pressures; Pintrich and De Groot, 1990; Zimmerman and Martinez-Pons, 1988). Existing studies have provided evidence supporting the positive association between intrinsic motivation and higher academic performance (Fong-Silva et al., 2018), whereas findings regarding the influence of extrinsic motivation are mixed, indicating both positive and negative effects depending on the context (Liu et al., 2024; Givens Rolland, 2012) Despite extensive research investigating motivation and academic achievement among general student populations, comparatively little is known about how these relation-ships manifest specifically among students from low-income families, a population facing unique motivational dynamics due to economic constraints (Ricketts et al., 2017).

### 1.3 Existing research gaps

Previous studies have predominantly utilized correlation and regression analyses to explore the association between motivation and academic performance (Kusurkar et al., 2012; Korpershoek et al., 2016; Ratelle et al., 2007), focusing largely on cross-sectional data (Pekrun et al., 2002; Wu et al., 2020). These analytical approaches limit our understanding of the complex interrelationships and potential mediating processes underlying the observed associations (Kocak et al., 2023; Green et al., 2012). Structural equation modeling (SEM) provides an effective statistical approach for examining these nuanced relationships, as it simultaneously evaluates direct and indirect pathways among multiple constructs, including potential mediators and latent variables (Wu et al., 2020). However, SEM-based analyses specifically focusing on low-income college students’ motivational dynamics and their academic outcomes remain scarce.

### 1.4 Theoretical framework and study contribution

Guided by self-determination theory and the transactional model of stress, the present study applies structural equation modeling (Mplus 8.3) to clarify how intrinsic motivation, extrinsic motivation and amotivation influence academic performance among low-income undergraduates. Specifically, we test two parallel mediating mechanisms—learning strategies and perceived stress—to disentangle the direct and indirect pathways from motivation to achievement (An et al., 2024; Garriott and Nisle, 2018). By extending motivational research to an economically disadvantaged cohort and incorporating stress as a negative mediator, the study refines extant theory and broadens empirical evidence.

### 1.5 Research objectives and hypotheses

The study pursues three objectives: (i) to determine whether intrinsic motivation positively predicts GPA; (ii) to examine whether amotivation exerts a negative influence; (iii) to explore whether extrinsic motivation affects GPA indirectly via learning strategies. We further test two mediators—learning strategies and perceived stress—that may transmit motivational effects. Accordingly, we propose:

*H1*: Intrinsic motivation positively predicts GPA.

*H2*: Amotivation negatively predicts GPA.

*H3*: Extrinsic motivation has no direct effect on GPA but yields a positive indirect effect through learning strategies.

*H4a*: Learning strategies mediate the links between all three motivation types and GPA.

*H4b*: Perceived stress mediates the links between all three motivation types and GPA.

**Figure 1:**
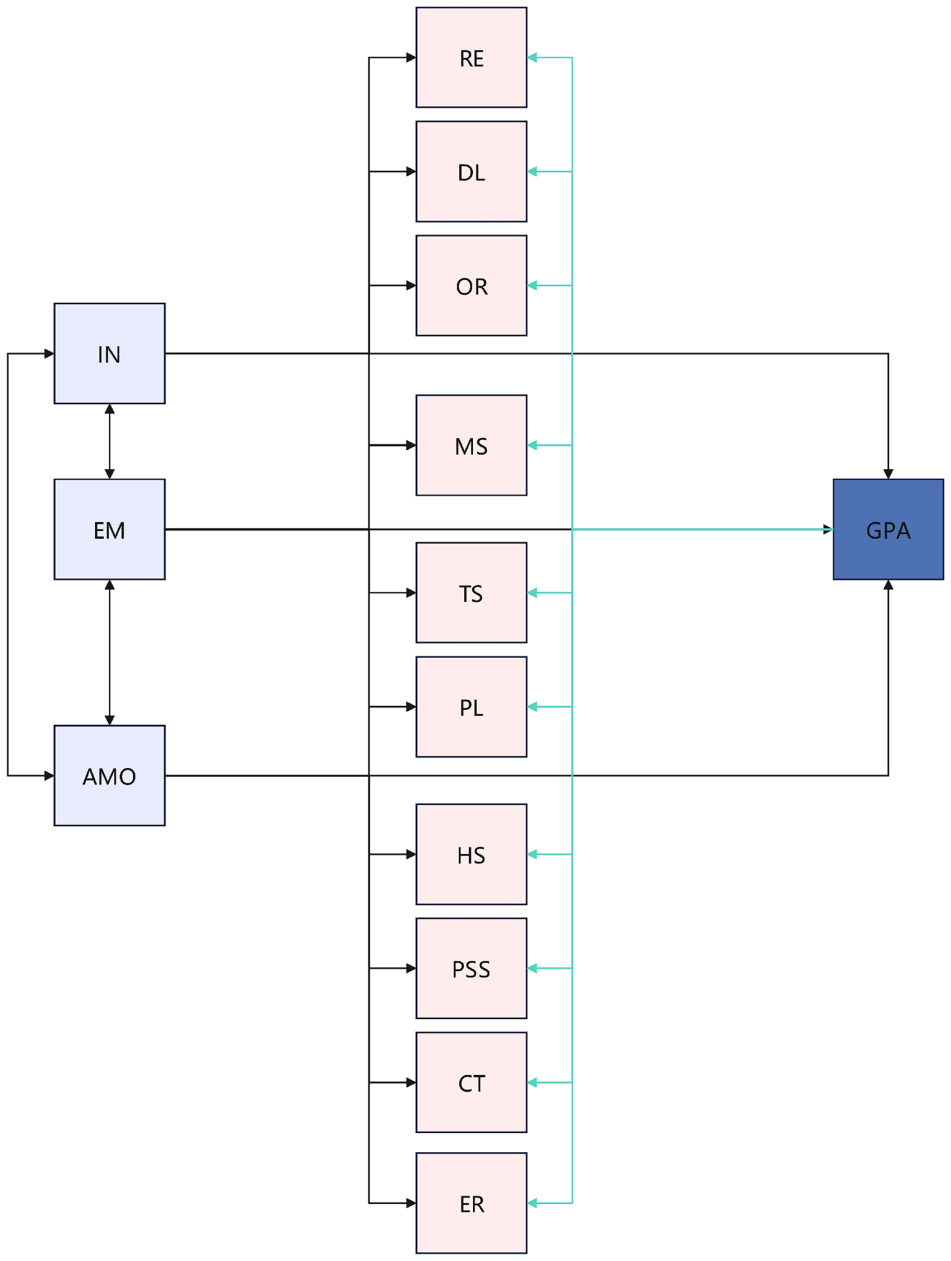
Hypothesized structural model.

The research path diagram structural equation model is shown in Figure 1.

### 1.6 Study implications

This study provides actionable insights for the participating universities, teaching staff, and related educational bodies. Universities should incorporate targeted programs—such as goal-setting workshops, peer mentoring, and recognition systems—that enhance intrinsic motivation and support self-regulated learning among low-income students. Teachers can adopt context-relevant instruction, systematic formative feedback, and activities that build metacognitive self-regulation, rehearsal, and critical thinking. At the institutional level, integrating academic strategy training with stress-management resources through coordinated efforts between departments, counseling services, and community organizations can address both cognitive and emotional barriers, helping narrow achievement gaps linked to economic disadvantage.

## 2 Materials and methods

### 2.1 Participants

A total of 316 full-time undergraduate students (56% female; M age = 19.7 ± 1.1 years) from three public universities in eastern China participated in this study. Participants were recruited using stratified random sampling, with strata defined by academic year (first to fourth year) and major discipline (social sciences, natural sciences, engineering), to ensure representation across academic backgrounds.

Eligibility criteria included: (i) official classification as “low-income” by the university’s student affairs department (based on provincial poverty threshold and documented family income); (ii) full-time enrollment during both data collection waves; and (iii) voluntary, informed consent.

To justify the sample size, we applied the Raosoft sample size calculator, assuming a 95% confidence level, 5% margin of error, and a total population of approximately 1,200 low-income undergraduates. The recommended minimum sample was 291, and the final sample of 316 exceeded this threshold, indicating adequate statistical power for structural equation modeling.

The effective response rate was 93.2%. Sample distribution by academic discipline was as follows: 38% social sciences, 34% natural sciences, and 28% engineering.

### 2.2 Measurement instruments

#### 2.2.1 Learning motivation

Learning motivation was assessed using the Academic Motivation Scale-College Version (AMS-C) developed by Vallerand et al. (1992). The AMS-C is a 28-item scale comprising three distinct dimensions: intrinsic motivation, extrinsic motivation, and amotivation. Responses were provided on a 7-point Likert scale ranging from 1 (completely disagree) to 7 (completely agree), with higher scores indicating higher levels of learning motivation. Previous studies have confirmed the AMS-C as a reliable and valid instrument for assessing motivation among Chinese college students (Liu et al., 2020; Zhang et al., 2016). In this study, Cronbach’s *α* coefficients for the overall scale and each dimension exceeded 0.80, indicating strong internal consistency.

#### 2.2.2 Learning strategies

Learning strategies were measured using the Motivated Strategies for Learning Questionnaire—Short Form (MSLQ-SF; Pintrich et al., 1993), translated and validated for Chinese higher-education contexts. For the present study, the instrument underwent a rigorous translation procedure. Two bilingual researchers independently translated the items from English to Chinese, and an independent bilingual expert performed back-translation. Discrepancies were resolved through consensus meetings, and the final Chinese version was reviewed by a panel of three educational psychology experts to ensure semantic equivalence and cultural appropriateness. All items are rated on a 7-point scale from 1 = “not at all like me” to 7 = “very much like me.” Subscale scores were computed as the mean of their constituent items after reverse-keyed statements had been recoded (reverse scoring: 8 − raw score). The instrument taps five strategy domains: rehearsal, elaboration, organization, critical thinking, and metacognitive self-regulation, plus three resource-management domains (time and study environment, effort regulation, peer learning/help seeking). Cronbach’s α coefficients for the eight domains ranged from 0.71 to 0.88.

#### 2.2.3 Psychological stress

Perceived stress is a critical psychological construct closely linked to well-being across diverse populations, including parents of children with developmental disabilities, where elevated stress can adversely affect overall psychological health (Putra et al., 2025). In the present study, perceived stress was conceptualized as a state-like, context-dependent construct, reflecting students’ subjective appraisal of academic and life stressors during the semester. This operationalization aligns with the transactional model of stress, which views stress as an ongoing interaction between the individual and their environment rather than as a fixed personality trait. In the present study, perceived stress was assessed using the 10-item Simplified Chinese version of the Perceived Stress Scale (SCPSS-10), which has demonstrated satisfactory psychometric properties in Chinese university students(Lu et al., 2017). The scale consists of six negatively worded items (e.g., “It feels hard to cope with all the things that must be done.”) and four positively worded items (e.g., “Have full confidence in one’s ability to handle personal issues.”), each rated on a 5-point Likert scale ranging from 0 (“never”) to 4 (“very often”). Following reverse coding of the positively keyed items, total scores range from 0 to 40, with higher scores indicating greater perceived stress. Exploratory and confirmatory factor analyses in prior validation research supported a stable two-factor structure, and the scale demonstrated good internal consistency (Cronbach’s *α* = 0.85) and acceptable test–retest reliability (*r* = 0.70) in a large sample of Chinese university students. In the present study, the internal consistency reliability was also acceptable (Cronbach’s *α* = 0.82), supporting the instrument’s applicability in this sample.

#### 2.2.4 Academic performance

Academic achievement was indexed by self-reported Grade Point Average (GPA) on the institution’s 0–4 scale. Extensive evidence indicates that self-reported GPA correlates strongly with transcript-verified records (Kuncel et al., 2005; Richardson et al., 2012).

### 2.3 Research program

The study received ethical approval from the Institutional Review Board of Heze University (approval number: HZU2024-001). All participants received a standardized briefing explaining the study’s aims, the voluntary nature of participation, and the confidentiality of their responses. Written informed consent was obtained prior to data collection. Ethical procedures were designed to comply with the Declaration of Helsinki, and particular care was taken to safeguard the rights and welfare of students from economically disadvantaged backgrounds. Data collection was carried out in two waves: Time 1 in September 2024 and Time 2 in March 2025. Prior to data administration, trained research staff explained the study purpose, confidentiality terms, and participation rights in group sessions.

Participant recruitment employed stratified random sampling across departments and year levels to ensure adequate coverage of the low-income student population. Screening was based on institutional records of household income, and only those officially certified as economically disadvantaged were invited to participate.

All data were collected using paper-based questionnaires administered in supervised classroom settings. Completed responses were stored in secure, locked archives until digital entry and analysis.

### 2.4 Statistical analysis methods

Analyses were conducted with SPSS 26.0 and Mplus 8.3. Descriptive statistics and Pearson correlations were computed in SPSS. Harman’s single-factor test indicated that common-method variance was within acceptable limits. Structural equation modeling (SEM) with maximum-likelihood estimation was performed in Mplus to test the hypothesized mediation model. Model evaluation relied on χ^2^, CFI, TLI, RMSEA, and SRMR. Bias-corrected bootstrap confidence intervals (1,000 resamples) were used to assess the significance of indirect effects.

## 3 Results

### 3.1 Descriptive statistical analysis

The final sample comprised 316 undergraduate students. Descriptive indices for all manifest variables fell within the theoretical response range (1–7 for Likert-type items, 0–4 for GPA). Means (M) and standard deviations (SD) were as follows: intrinsic motivation (IM) *M* = 3.97, SD = 0.66; extrinsic motivation (EM) *M* = 3.97, SD = 0.60; amotivation (AMO) *M* = 3.96, SD = 0.88; GPA *M* = 2.77, SD = 0.47. Learning-strategy subscales showed moderate central tendencies (*M* = 3.27–3.80) with SDs between 0.60 and 0.99, while perceived stress (PSS) was comparatively low (*M* = 1.99, SD = 0.33). All variables displayed acceptable skewness (│Sk│ < 0.22) and kurtosis (│Ku│ < 0.88), supporting the use of maximum-likelihood estimation (Table 1).

**Table 1:** Descriptive statistics and normality test of research variables.

Variable	Mean	SD	Skewness	Kurtosis
IM	3.971	0.665	0.037	−0.629
EM	3.971	0.6	0.046	−0.273
AMO	3.958	0.878	0.012	−0.41
RE	3.436	0.896	0.215	−0.037
EL	3.656	0.978	0	−0.385
OR	3.466	0.974	0.048	−0.052
CT	3.606	0.892	0.207	0.468
MS	3.601	0.888	0.122	−0.276
TS	3.545	0.694	0.109	−0.512
ER	3.681	0.974	0.133	−0.288
PL	3.267	0.988	−0.041	−0.606
HS	3.803	0.597	−0.183	−0.343
PSS	1.992	0.333	−0.03	−0.88
GPA	2.768	0.474	0.021	−0.639

### 3.2 Structural model fit

Within a path framework that included both direct and indirect routes, overall fit was acceptable (*χ*^2^ = 75.11, df = 45, *p* = 0.003; RMSEA = 0.046, 90% CI = 0.027–0.064, pRMSEA ≤ 0.05 = 0.62; CFI = 0.980; TLI = 0.961; SRMR = 0.036). These values meet widely accepted criteria for close approximation, indicating that the proposed pattern of relations reproduces the observed covariance structure satisfactorily (Table 2).

**Table 2:** Overall goodness of fit indicators for the intermediary structure model.

Fit index	Value
*χ* ^2^	75.114
df	45
*p*-value	0.0032
RMSEA	0.046
RMSEA 90% CI	0.027–0.064
CFI	0.98
TLI	0.961
SRMR	0.036
AIC	6487.622
BIC	6731.745
SSA-BIC	6525.581

### 3.3 Structural path analysis

Standardized path coefficients (STDYX) revealed that GPA was positively and directly predicted by intrinsic motivation (*β* = 0.205, SE = 0.052, *z* = 3.96, *p* < 0.001) and negatively by amotivation (*β* = −0.240, SE = 0.047, *z* = −5.12, *p* < 0.001) and perceived stress (*β* = −0.192, SE = 0.033, *z* = −5.84, p < 0.001). Extrinsic motivation showed no direct relation to GPA (*β* = −0.019, *p* = 0.611). Among the learning-strategy indicators, metacognitive self-regulation (MS; *β* = 0.114, *p* = 0.015), critical thinking (CT; *β* = 0.091, *p* = 0.036), and rehearsal (RE; *β* = 0.096, *p* = 0.013) contributed uniquely to attainment, whereas other strategy paths were nonsignificant (|β| ≤ 0.08, *p* > 0.05). The model accounted for 64.3% of the variance in GPA (*R*^2^ = 0.643) (Table 3).

**Table 3:** Standardized direct effects of predictors on GPA.

Predictor	*β*	SE	*p*
IM	0.205	0.052	<0.001
EM	−0.019	0.037	0.611
AMO	−0.24	0.047	<0.001
RE	0.096	0.039	0.013
EL	0.053	0.047	0.263
OR	0.037	0.043	0.396
CT	0.091	0.043	0.036
MS	0.114	0.047	0.015
TS	0.08	0.043	0.065
ER	0.078	0.045	0.083
PL	0.011	0.041	0.797
HS	0.056	0.04	0.17
PSS	−0.192	0.033	<0.001

### 3.4 Predictors of learning strategies and perceived stress

All nine learning-strategy facets were consistently enhanced by intrinsic motivation (*β* = 0.274–0.435, all *p* < 0.001) and suppressed by amotivation (*β* = −0.162 to −0.358, all *p* ≤ 0.008). Extrinsic motivation exerted smaller positive effects (*β* = 0.104–0.190) that reached significance on seven of the nine facets (*p* ≤ 0.022). Perceived stress was lower for students high in intrinsic motivation (*β* = −0.164, *p* = 0.007) and higher for those high in amotivation (*β* = 0.142, *p* = 0.019); the EM → PSS path was null (Table 4).

**Table 4:** The standardized path coefficients of motivational variables on learning strategies and psychological stress.

Strategy/PSS	IMβ	EMβ	AMOβ
RE	0.354	0.139	−0.285
EL	0.415	0.119	−0.337
OR	0.364	0.148	−0.238
CT	0.366	0.129	−0.329
MS	0.435	0.127	−0.358
TS	0.383	0.124	−0.296
ER	0.382	0.104	−0.299
PL	0.392	0.19	−0.174
HS	0.274	0.166	−0.162
PSS	−0.164	−0.014	0.142

### 3.5 Indirect and total effects on GPA

**Figure 2:**
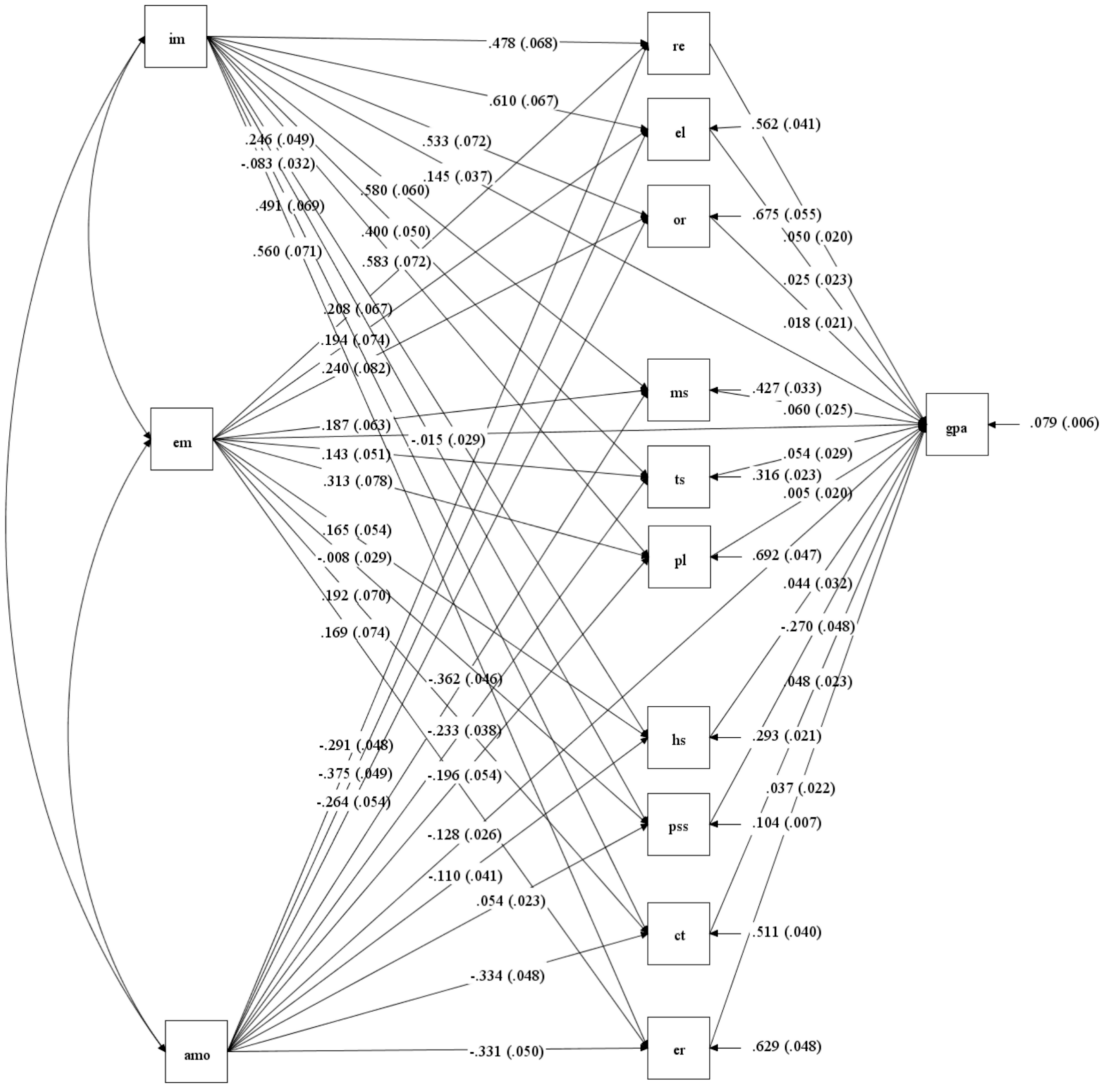
Path diagram of the structural model.

Indirect and total effects on GPA shown in Table 5 and Figure 2.

**Table 5:** GPA the direct effect, indirect effect and total effect of GPA.

Motivation	Direct *β*	Total indirect *β*	Total *β*	Direct *p*	Indirect *p*	Total *p*
IM	0.205	0.263	0.468	<0.001	<0.001	<0.001
EM	−0.019	0.083	0.064	0.611	<0.001	0.092
AMO	−0.24	−0.209	−0.449	<0.001	<0.001	<0.001

## 4 Discussion

### 4.1 Intrinsic motivation enhances GPA among students

Consistent with Hypothesis 1, we found that intrinsic motivation contributes to higher GPA through both direct and indirect pathways. The direct pathway was statistically reliable, while the indirect pathway—operating via enhanced learning strategies and reduced perceived stress—was relatively larger (Hauzel et al., 2024). This pattern accords with self-determination theory, which holds that autonomous motivation sustains engagement, deepens cognitive processing, and promotes adaptive stress appraisal. Our analysis on low-income undergraduates indicated that those with stronger intrinsic motivation engaged more in metacognitive self-regulation (MS), rehearsal (RE), and critical thinking (CT), while also reporting lower stress levels. This combination appears particularly advantageous under financial strain, as perceived control and sustained engagement can help buffer against academic disruption (Van Beuningen, 2024). Taken together, these results support an integrative motivational–stress perspective in which motivational regulation influences achievement partly through stress-appraisal processes, with autonomous motivation fostering higher perceived control and adaptive coping, thereby lowering stress, whereas controlled or amotivated states may heighten perceived demands, reduce coping efficacy, and undermine performance. Overall, these findings underscore that intrinsic motivation not only directly supports achievement but also strengthens resilience against stress, making it a pivotal driver of academic success under financial hardship.

### 4.2 Mechanisms underlying the negative impact of Amotivation

Hypothesis 2 was supported: amotivation showed the strongest overall negative association with GPA, via both a direct pathway and an additional pathway through lower use of learning strategies and higher stress. A sizable portion of the overall association was mediated by these mechanisms (Vlachopanou et al., 2025; Gordeeva et al., 2024). These results are consistent with expectancy–value theory, which suggests that when perceived competence and task value are low, students allocate less effort, display diminished persistence, and become more vulnerable to stress-related disengagement. In resource-constrained environments, such disengagement can be magnified by heightened threat-oriented appraisals, exacerbating both stress and performance decline (Manzano-Sánchez, 2023). This evidence underscores the importance of identifying and addressing amotivation early, particularly for students facing structural disadvantages. By mitigating amotivation’s dual impact on strategy use and stress, targeted interventions could substantially reduce its detrimental influence on academic outcomes.

### 4.3 How extrinsic motivation improves GPA indirectly

Hypothesis 3 received partial support. Extrinsic motivation was unrelated to GPA through a direct pathway (Bin Abdulrahman et al., 2023) but exhibited a small, statistically reliable indirect association via learning strategies (Li et al., 2023), yielding a total association that was not distinguishable from zero. In practical terms, disciplined behaviors linked to external incentives can support achievement when they translate into strategic studying, yet they do not appear to alleviate stress on their own. This pattern complements prior work suggesting that controlled forms of motivation guide performance-oriented behaviors but are less effective in promoting resilience and stress management. Thus, while extrinsic motivation can facilitate disciplined study habits, its limited role in stress regulation constrains its overall contribution to academic achievement.

### 4.4 Learning strategies and perceived stress as parallel mediators

Hypothesis 4a was supported, and Hypothesis 4b received partial support, clarifying the dual processes linking motivation to academic performance. Learning strategies mediated the effects of all three motivational constructs, with metacognitive self-regulation, rehearsal, and critical thinking uniquely predicting GPA, while other strategies did not show unique predictive value in this context. Perceived stress operated as a parallel mediator for intrinsic motivation and amotivation—but not for extrinsic motivation—aligning with an integrated self-regulation–stress framework in which cognitive regulation and emotional appraisal exert independent and additive influences on performance (Goetz et al., 2020). These findings highlight the value of interventions that concurrently strengthen strategic learning and reduce maladaptive stress appraisal. Ntegrating cognitive and emotional regulation training may therefore yield the most robust gains in academic performance across diverse student populations.

## 5 Conclusion

This study demonstrates that among low-income undergraduates, the quality of learning motivation plays a decisive role in shaping academic outcomes. Intrinsic motivation emerged as the most powerful positive predictor of GPA, exerting both a direct effect and an indirect influence through enhanced metacognitive self-regulation, rehearsal, and critical thinking, alongside reduced perceived stress. Amotivation constituted the most detrimental factor, impairing achievement via diminished strategic learning behaviors and elevated stress levels. Extrinsic motivation, by contrast, conferred only modest benefits, operating exclusively through incremental improvements in strategy use and without any direct association with GPA. Learning strategies and perceived stress functioned as independent, parallel mediators, underscoring the need for interventions that integrate cognitive skills training with stress-management resources. The integrated model accounted for 64% of the variance in GPA, offering a robust empirical foundation for multifaceted institutional policies aimed at reducing achievement gaps for students under financial strain. These findings suggest that sustainable improvement in academic performance requires fostering autonomous motivation, providing targeted support for strategic learning, and addressing the emotional burdens associated with economic disadvantage. While the present results are context-specific, they contribute to a broader understanding of how motivational quality and self-regulation jointly influence achievement, with implications for global efforts to enhance equity in higher education.

### 5.1 Limitations and future directions

Several constraints temper the generalizability of these findings. All variables relied on self-reports, raising the possibility of common-method bias; future work should integrate objective behavioral analytics (e.g., learning management system logs) and physiological stress markers. The sample derived from a single cultural context; cross-cultural replication would clarify the boundary conditions of the observed paths, particularly for extrinsic motivation. Additionally, unmeasured covariates such as prior academic ability or socioeconomic background might confound effect sizes. Incorporating multilevel modeling with classroom-level autonomy support, or testing moderated mediation by gender and major, would further enrich the explanatory framework.

## Data Availability

The original contributions presented in the study are included in the article/supplementary material, further inquiries can be directed to the corresponding author.
